# HMBA Releases P-TEFb from HEXIM1 and 7SK snRNA via PI3K/Akt and Activates HIV Transcription

**DOI:** 10.1371/journal.ppat.0030146

**Published:** 2007-10-12

**Authors:** Xavier Contreras, Matjaz Barboric, Tina Lenasi, B. Matija Peterlin

**Affiliations:** Departments of Medicine, Microbiology, and Immunology, Rosalind Russell Medical Research Center, University of California San Francisco, San Francisco, California, United States of America; The Salk Institute for Biological Studies, United States of America

## Abstract

Hexamethylene bisacetamide (HMBA) is a potent inducer of cell differentiation and HIV production in chronically infected cells. However, its mechanism of action remains poorly defined. In this study, we demonstrate that HMBA activates transiently the PI3K/Akt pathway, which leads to the phosphorylation of HEXIM1 and the subsequent release of active positive transcription elongation factor b (P-TEFb) from its transcriptionally inactive complex with HEXIM1 and 7SK small nuclear RNA (snRNA). As a result, P-TEFb is recruited to the HIV promoter to stimulate transcription elongation and viral production. Despite the continuous presence of HMBA, the released P-TEFb reassembles rapidly with 7SK snRNA and HEXIM1. In contrast, a mutant HEXIM1 protein that cannot be phosphorylated and released from P-TEFb and 7SK snRNA via the PI3K/Akt pathway antagonizes this HMBA-mediated induction of viral production. Thus, our studies reveal how HIV transcription is induced by HMBA and suggest how modifications in the equilibrium between active and inactive P-TEFb could contribute to cell differentiation.

## Author Summary

The reservoir of HIV in infected people remains an insurmountable problem in the era of highly active antiretroviral therapy. Thus, the virus persists despite the best possible treatment. HIV hides in many cells and tissues, where its genome is not expressed. Thus, neither drugs nor the immune system can eradicate it from the body. One hope is to activate the production of HIV in these reservoirs in the presence of optimal treatment. Strategies aimed at activating hematopoetic cells and thus viral replication have been tried and failed. In this report, we targeted a specific host transcriptional complex that is essential for the transcription of HIV genome. Its activation should not lead to generalized stimulation of the immune system. Indeed, paradoxically, hexamethylene bisacetamide (HMBA) and related compounds lead to cellular differentiation and apoptosis. By studying properties of these differentiation agents, we discovered that they activate transiently transcription of HIV, be it in stable cell lines or in primary infected cells. Thus, compounds related to HMBA, some of which have now been approved for clinical use, could be tried to diminish or eliminate the reservoir of HIV in optimally treated infected individuals.

## Introduction

Highly active antiretroviral therapy (HAART) has proven effective against progression to AIDS. Indeed, the viral loads can be lowered to undetectable levels in peripheral blood of HIV-infected individuals with this treatment. However, the persistence of latently infected cells in these patients prevents their cure. Indeed, these cells harbor integrated proviral genomes, which are insensitive to HAART and can be reactivated upon treatment interruption. Thus, one of the major therapeutic goals is to purge these latent reservoirs of HIV.

Proviral latency is established predominantly at the level of transcription [1,2]. Reactivating viral replication should render HIV susceptible to HAART and immune elimination. To this end, initial attempts included treatments with growth factors such as IL-2 or the activation of T cells with anti-CD3 antibodies, which failed to eradicate HIV and resulted in deleterious side effects [3,4]. Therefore, alternative approaches towards the reactivation of HIV must be developed. They should not induce a global stimulation of lymphocyte proliferation but activate specifically HIV transcription. Of note, prostratin, a compound that activates protein kinase C (PKC) and NF-κB [5,6], as well as IL-7, a key factor in lymphocyte homeostasis [7], can activate HIV transcription. In addition, the inhibition of histone deacetylases (HDACs), whose recruitment to the HIV promoter has been associated with transcriptional repression [8], can also activate viral transcription in peripheral blood mononuclear cells (PBMCs) from HAART-treated patients using valproic acid [9]. However, this compound is a weak HDAC inhibitor and despite encouraging results obtained in four patients [10], the latent reservoir was not reduced in patients receiving this drug chronically for neurological conditions [11].

Interestingly, hexamethylene bisacetamide (HMBA), which is a hybrid bipolar compound that induces terminal differentiation and apoptosis in transformed cells in culture [12,13], reactivates viral production in chronically infected cell lines [14,15]. This activation occurs at the level of transcription and is independent of NF-κB but requires Sp1-binding sites in the HIV promoter [15]. However, the mechanism by which HMBA induces HIV transcription remains unknown. One possible mechanism could involve increased DNA accessibility and induction of nucleosome remodeling [16]. However, HMBA neither inhibits HDACs nor increases histone acetylation [17]. Alternatively, HMBA could mediate its effects on viral transcription via the activation of cellular kinases. Indeed, PKC and calcium pathways are activated by HMBA [18]. In addition, suberoylanilide hydroxamic acid (SAHA), a bipolar compound that is structurally similar to HMBA, activates Akt [19,20]. Importantly, HMBA increases greatly the expression of HMBA-induced protein 1 (HEXIM1) [21,22] and its homolog HEXIM2, which, in concert with 7SK small nuclear RNA (snRNA), inhibit and sequester the positive transcription elongation factor b (P-TEFb) in its transcriptionally inactive complex (large complex [LC]) [23–28]. Besides the LC, P-TEFb, which is composed of cyclin-dependent kinase 9 (Cdk9) and cyclin T1 (CycT1), predominantly binds Brd4 [29,30] or is in a free heterodimeric form (small complex [SC]). As such, P-TEFb is transcriptionally active and can be recruited to the HIV promoter to stimulate viral transcription elongation by phosphorylating the C-terminal domain of RNA polymerase II (RNAPII) and negative transcription elongation factors [31,32]. Thus, the exposure of cells to HMBA leads to two seemingly opposite phenotypes, the induction of HIV transcription and increased levels of HEXIM1 and HEXIM2. However, it is possible that these events do not occur simultaneously. Indeed, the partitioning of P-TEFb into inactive and active complexes is dynamic, and several stress-inducing agents disrupt the LC and result in the release of P-TEFb [23,30,33]. Of note, in the course of our studies, it was demonstrated that HMBA also disrupts the LC, which resulted in cellular differentiation [34]. However, the nature of this disruption remains unknown.

In this study, we investigated the mechanism by which HMBA induces viral production. We found that HMBA activates Akt transiently via the phosphatidylinositol-3-kinase (PI3K), leading to the concomitant disruption of the LC and the recruitment of P-TEFb to the HIV promoter. Importantly, the inhibition of the PI3K/Akt pathway in chronically infected cell lines or the expression of a mutant HEXIM1 protein that could not be phosphorylated by Akt, and was resistant to HMBA-mediated disruption of the LC, antagonized the induction of HIV transcription by HMBA. Thus, our studies reveal how HMBA releases P-TEFb to reactivate viral production from latency.

## Results

### HMBA Induces Viral Production in Chronically Infected Cell Lines and Resting CD4+ T Cells

**Figure 1:**
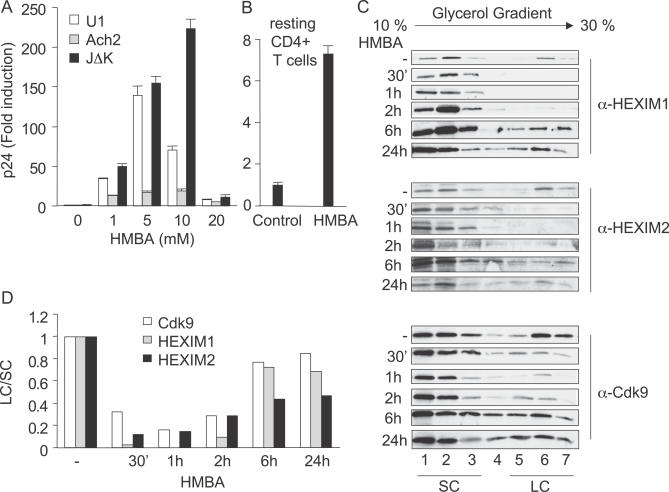
HMBA Transiently Disrupts the LC (A) U1, Ach-2, and JΔK cells were stimulated with increasing concentrations of HMBA (1, 5, 10, and 20 mM) and viral production was assessed at 48 h in the supernatant using p24 ELISA. (B) Resting CD4+ T cells, which were isolated from PBMCs that were infected with HIV-1_LAI_ and rested for 11 d were treated or not with HMBA (1 mM) for 3 d. Viral production was assessed using p24 ELISA. (C) Total cell lysates of Jurkat cells stimulated or not with HMBA (5 mM) for 30 min, 1 h, 2 h, 6 h, and 24 h, were subjected to glycerol gradient sedimentation analyses (10%–30%), and the fractions were analyzed by western blotting using HEXIM1, HEXIM2, and Cdk9 antibodies. Numbers below the western blots correspond to fractions from glycerol gradient analyses. (D) LC/SC represents the ratio of cumulated intensities of fractions 5, 6, and 7 (LC) over intensities of fractions 1, 2, and 3 (SC). Values were normalized to ratios obtained in unstimulated cells.

Since chronically infected U1 (monocytic lineage), ACH-2, and JΔK (lymphocytic lineages) cells represent model systems for proviral latency in the host, we first examined effects of HMBA on the production of HIV particles in these cells (Figure 1A). Cells were stimulated with increasing concentrations of HMBA and the release of viral particles was measured by p24 ELISA after 24 h in their supernatants. As expected, HMBA induced viral production in a dose-dependent manner in all cells examined (Figure 1A). At the optimal concentration of HMBA, viral production in U1, ACH-2, and JΔK cells increased by 135-, 25-, and 220-fold, respectively. Since viral production increased the most in JΔK cells that bear an integrated HIV genome lacking two NF-κB binding sites, this stimulatory effect of HMBA did not depend on NF-κB. At higher concentrations, HMBA was less efficient at inducing viral production, most likely due to its pronounced cytotoxic effects as determined by trypan blue staining and FACS analyses (not shown). In addition, HMBA induced an increase of the luciferase activity from HeLa cells that contained an integrated luciferase gene under the control of the HIV promoter (not shown). We conclude that HMBA induces viral production in chronically infected cell lines, most likely through an NF-kB-independent activation of HIV transcription.

To confirm these effects on viral production in primary cells, peripheral blood lymphocytes (PBLs) were activated for 3 d, infected with HIV-1_LAI_, and rested for 11 d. Next, resting CD4+ T cells were isolated using anti-CD4 magnetic beads and washed extensively. By FACS, these cells expressed neither CD25 nor HLA-DR (not shown). Before the addition of HMBA, we observed few if any viral particles in the supernatants of these selected cells (Figure 1B, control). In contrast, in the presence of HMBA, levels of p24 increased 7-fold in their supernatants (Figure 1B, HMBA). Thus, HMBA also activates viral production in resting CD4+ T cells.

### HMBA Disrupts Transiently the LC

Previous studies established that HMBA activates HIV transcription [14,15,34]. Moreover, HIV gene expression depends critically on the kinase activity of P-TEFb [31]. Finally, HMBA induces the expression of HEXIM1 and HEXIM2, which together with 7SK snRNA inhibit P-TEFb in the LC [35]. Thus, HMBA could disrupt the LC, albeit transiently, to activate P-TEFb for the stimulation of HIV transcription. Therefore, we analyzed the LC and SC by glycerol gradient sedimentation analyses with anti-HEXIM1, HEXIM2, and P-TEFb antibodies at various times in Jurkat cells that were treated with HMBA (Figure 1C). As described previously, the presence of HEXIM1/2, CycT1, and Cdk9 in high molecular weight fractions is characteristic of their presence in the LC with 7SK snRNA [25]. As presented in Figure 1C, HMBA induced the disruption of the LC (fractions 5, 6, and 7), starting as soon as 30 min and reaching a maximum at 1 h after the addition of HMBA. Since HEXIM1, HEXIM2, and Cdk9 reassociated into the LC as soon as 6 h after the addition of HMBA, this disruption was transient. Notably, the reassociation of P-TEFb with HEXIM1 was more efficient than the reassociation with HEXIM2. Also, whereas overall amounts of HEXIM1 and HEXIM2 increased after 6 h of stimulation, levels of Cdk9 did not change significantly. Quantitative data presented in Figure 1D demonstrate that ratios between LC and SC (LC/SC) were minimal at 1 h and came back to pre-treatment values at 6 h post-stimulation. Thus, whereas HMBA disrupts the LC transiently in Jurkat cells, a prolonged exposure to this compound leads to the establishment of a new equilibrium between LC and SC.

### HMBA Induces Viral Production via the Activation of PI3K and Akt

**Figure 2:**
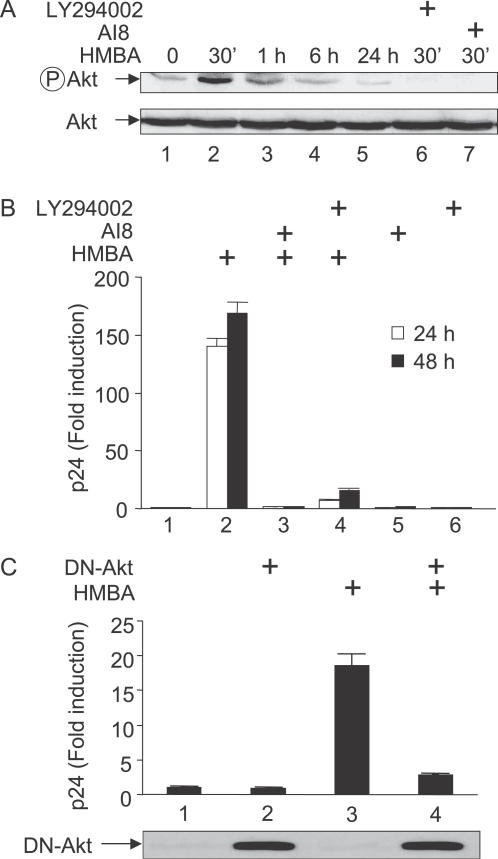
HMBA Induces HIV Production from Latently Infected Cell Lines via the PI3K/Akt Pathway (A) Total cell lysates of Jurkat cells preincubated or not with inhibitors of Akt or PI3K (AI8 or LY294002) and stimulated or not by HMBA (5 mM) for 30 min, 1 h, 6 h, and 24 h were analyzed by western blotting using anti-phospho-Akt antibodies, or Anti-Akt antibodies for loading controls. (B) U1 cells were cultured in the presence or absence of LY294002 (10 μM) or AI8 (1 μM) prior to stimulation with HMBA (5 mM). Concentrations of p24 antigens were then measured from supernatants at 24 and 48 h after stimulation to assess viral production. (C) U1 cells were transfected with a plasmid expressing a dominant negative mutant Akt protein (DN-Akt) or with an empty plasmid vector. After 24 h, cells were stimulated or not with HMBA (5 mM) and viruses in the supernatant were quantified 24 h later using p24 ELISA. Lower panels represent the levels of DN-Akt expressed in cells as assessed using glu-glu tag antibodies.

To determine the mechanism by which HMBA induced such a rapid, transient disruption of the LC, we reasoned that HMBA could act by activating cellular signaling pathways. Since SAHA is similar in structure to HMBA [13,17] and it activates the PI3K/Akt pathway [20], we hypothesized that HMBA could do the same. Indeed, HMBA activated Akt transiently in Jurkat cells as determined with an antibody that recognizes the active, phosphorylated form of Akt (Figure 2A). This activation which was detected after 30 min of stimulation, decreased significantly at 6 h (Figure 2A, lanes 2–4). Moreover, 24 h later, levels of activated Akt were even lower than those in untreated cells (compare lanes 1 and 5). Importantly, HMBA-mediated activation of Akt was inhibited when cells were pre-incubated with the PI3K inhibitor LY294002 (Figure 2A, lane 6) or the Akt inhibitor 8 (AI8, Figure 2A, lane 7). Thus, similar to SAHA, HMBA induces a transient activation of Akt via PI3K and results in a long-term inhibition of this signaling pathway. Importantly, the kinetics of Akt activation correlated nicely with the kinetics of disruption of the LC.

To investigate this activation of Akt further, we next asked whether these kinases were required for the stimulatory effect of HMBA on viral production. We pre-incubated U1 cells with AI8 or LY294002 prior to the stimulation with HMBA and measured viral production as in Figure 1A. In these cells, HMBA stimulated viral production by approximately 150-fold (Figure 1A, lane 2). Critically, in U1 cells, inhibitors of PI3K and Akt reduced the HMBA-induced viral production by 97% and 90%, respectively (Figure 2B, lanes 3 and 4). In contrast, these inhibitors had only minor effects on basal levels of viral production (lanes 5 and 6). Consistent with these results, the expression of a mutant dominant negative form of Akt (DN-Akt) also prevented the stimulation of viral production by HMBA in U1 cells (Figure 2C, compare lanes 3 and 4). In addition, similar effects were observed in ACH-2 and JΔK cells (not shown). Taken together, these results demonstrate that a transient activation of the PI3K/Akt pathway is required for the HMBA-induced viral production in chronically infected cell lines.

### HMBA Disrupts the LC via the Activation of the PI3K/Akt Pathway

**Figure 3:**
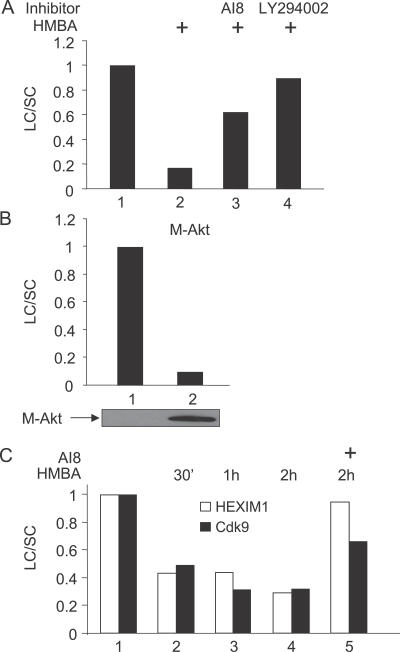
HMBA Disrupts the LC via the PI3K/Akt Pathway Jurkat cells were stimulated or not with HMBA (5 mM) for 1 h in the presence or absence of LY294002 (10 μM) or AI8 (1 μM) in (A) or were electroporated with the empty plasmid vector or a vector expressing M-Akt for 24 h in (B). Total cell lysates were then subjected to glycerol gradient sedimentation and fractions were analyzed as in Figure 1. Western blotting was performed using anti-HEXIM1 antibodies. Results present the ratio LC/SC as in Figure 1. In (C), a similar analysis was performed using total cell lysates of non-adherent PBMCs pre-treated or not with AI8 and stimulated or not with HMBA (5 mM) for 30 min, 1 h, and 2 h.

Thus far, our results indicated that HMBA disrupts the LC transiently and that it induces viral production via PI3K and Akt. To investigate whether the disruption of the LC by HMBA was also dependent on the PI3K/Akt pathway, we pre-incubated Jurkat cells with AI8 or LY294002 prior to the stimulation with HMBA and followed the partitioning of endogenous HEXIM1, HEXIM2, and P-TEFb in the LC and SC by glycerol gradient sedimentation analyses as in Figure 1C. Critically, both inhibitors prevented the HMBA-mediated disruption of the LC (Figure 3A, lanes 3 and 4). Similarly, when we expressed a mutant constitutively active form of Akt (M-Akt), the LC was also disrupted (Figure 3B). Finally, in addition to experiments performed in transformed cell lines, we also examined effects of HMBA in primary lymphocytes. As presented in Figure 3C, HMBA had the same effect on partitioning of HEXIM1 and Cdk9 in LC and SC in these cells. Moreover, AI8 also prevented the HMBA-induced disruption of the LC. Taken together, these results demonstrate that HMBA disrupts the LC via the activation of the PI3K/Akt pathway in transformed cell lines and in primary cells. Moreover, results from primary cells suggest that the disruption of the LC is likely to be critical for the HMBA-induced viral production in vivo.

### HMBA Induces the Recruitment of P-TEFb to the HIV Promoter and Promotes Transcription Elongation

**Figure 4:**
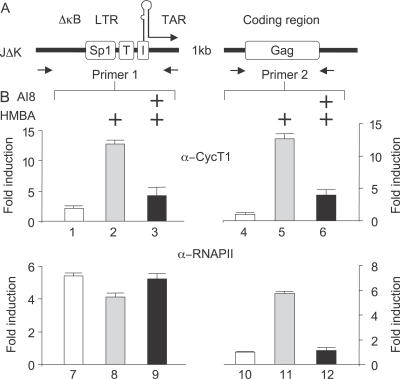
HMBA Induces the Recruitment of P-TEFb to the HIV Promoter (A) Schematic representation of the HIV promoter (LTR) and coding region (Gag). Lack of NF-κB sites is denoted by ΔκB. LTR: long terminal repeat; Sp1: 3 Sp1 binding sites; T: TATA box; I: initiator; TAR, transactivation response element; Gag: group specific antigen. (B) JΔK cells, chronically infected with HIV-1 lacking NF-κB binding sites, were pre-treated or not with AI8 prior to the stimulation with HMBA (5 mM, 1 h) and proteins were fixed onto DNA with formaldehyde. Following sonication, the anti-CycT1 or anti-RNAPII antibodies were added to the chromatin solution for immunoprecipitation of DNA–protein complexes. PCR was performed with the indicated primers (A) to analyze amounts of DNA that were associated with CycT1 or RNAPII at the promoter (primer 1) or 1,000 nt downstream in the Gag gene (primer 2). Quantitative PCRs were performed with anti-RNAPII or anti-CycT1-immunoprecipitated samples, as well as with input DNA before immunoprecipitation, which served as controls for the amplification efficiency of individual sets of PCR primers. Immunoprecipitates obtained without using any antibody were used as negative controls.

Since HMBA disrupted the LC and thus released active P-TEFb, we wanted to determine if the released SC could then be recruited to the HIV promoter, leading to increased transcription elongation of the viral genome. To test this hypothesis, we followed occupancies of P-TEFb and RNAPII on the HIV promoter and the downstream coding sequences by performing chromatin immunoprecipitation assays (ChIPs; Figure 4). Positions of primers used in our ChIPs are depicted in Figure 4A. First, we stimulated JΔK cells with or without HMBA for 1 h in the presence or absence of AI8 prior to ChIPs. To detect P-TEFb and RNAPII, we used antibodies directed against CycT1 and the large subunit of RNAPII. Consistent with our findings that HMBA disrupts the LC, P-TEFb was recruited efficiently to the HIV promoter and coding region only upon the stimulation with HMBA, whereas this event was inhibited in the presence of AI8 (Figure 4B, top panels). Moreover, whereas levels of RNAPII increased markedly on the coding region in the presence of HMBA, AI8 prevented this effect (Figure 4B, bottom panel). Notably, HMBA stimulation resulted in a slightly decreased occupancy of RNAPII on the HIV promoter, possibly reflecting increased rates of transcription elongation. In contrast, the presence of AI8 abolished this decrease. We conclude that HMBA disrupts the LC in a PI3K/Akt-dependent manner to release P-TEFb that is then recruited to the HIV promoter and coding sequences. Consequently, RNAPII elongates successfully on the viral genome. Moreover, as these findings were obtained in JΔK cells that contained an integrated HIV provirus lacking NF-κB binding sites, these results suggest further that the stimulation of HIV transcription by HMBA can occur independently of NF-κB.

### Conserved Threonine and Serine Residues in the CycT-Binding Domain of HEXIM1 Are Critical for P-TEFb Binding

**Figure 5:**
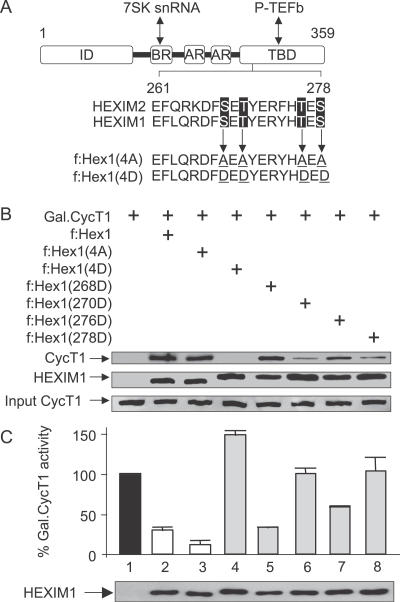
Mutant f:Hex1(4D) Protein Is Functionally Inactive (A) Schematic representation of HEXIM1. Sequences are representative of mutant f:Hex1(4A) and f:Hex1(4D) proteins. Mutations are underlined. ID: inhibitory domain; BR: basic region; AR: acidic region; TBD, CycT-binding domain. (B) Extracts prepared from Jurkat cells transfected with an empty plasmid vector or those expressing f:Hex1, mutant f:Hex1(4A), or f:Hex1(4D) proteins, or point mutant f:Hex1(268D), f:Hex1(270D), f:Hex1(276D), or f:Hex1(278D) proteins, were subjected to anti-Flag immunoprecipitation. Levels of endogenous CycT1 protein bound to Flag epitope-tagged mutant HEXIM1 proteins are indicated in the upper panel. The middle panel represents levels of Flag epitope-tagged mutant HEXIM1 proteins expressed in Jurkat cells and the lower panel shows the input of CycT1. (C) Jurkat cells were co-transfected with the reporter plasmid pG6TAR (0.4 μg) and, where indicated, with the plasmids expressing Gal4.CycT1 (0.6 μg), f:Hex1 (0.8 μg), f:Hex1(4A) (0.8 μg), f:Hex1(4D) (0.8 μg), f:Hex1(268D) (0.8 μg), f:Hex1(270D) (0.8 μg), f:Hex1(276D) (0.8 μg), or f:Hex1(278D) (0.8 μg). Error bars represent the mean +/− SD. Lower panel presents the levels of Flag epitope-tagged HEXIM1 proteins expressed in Jurkat cells.

To demonstrate formally that observed effects of HMBA were mediated via the disruption of the LC, we next wanted to gain insight into the mechanism of this process. Importantly, when present in the LC, the CycT1 subunit of P-TEFb binds HEXIM1 directly via its CycT-binding domain (TBD) [33]. Since the activation of the PI3K/Akt pathway is required for the disruption of the LC, we reasoned that an active cellular kinase might target conserved residues in HEXIM1, whose phosphorylation could abrogate the binding between HEXIM1 and P-TEFb. Indeed, an inspection of the primary TBD sequences in HEXIM1 and HEXIM2 from different species identified a cluster of closely positioned serine and threonine residues that could be targeted by the PI3K/Akt pathway (Figure 5A). In HEXIM1, these included serines at positions 268 (S268) and 278 (S278) and threonines at positions 270 (T270) and 276 (T276). Next, mutant Flag epitope-tagged HEXIM1 proteins were generated, in which these four residues were replaced by alanines (f:Hex1(4A)) or aspartic acid (f:Hex1(4D)), individually or in combination. Since alanines cannot be phosphorylated, we reasoned that some of these mutant HEXIM1 proteins should resist HMBA-mediated disruption of the LC and should antagonize its effects in cells. On the other hand, since aspartic acid mimics constitutively phosphorylated residues, some of these mutant f:Hex1 proteins should fail to bind P-TEFb in the LC.

First, we analyzed the binding and inhibitory properties of these mutant f:Hex1 proteins in cells (Figure 5B and 5C). To examine their binding to P-TEFb, wild-type and mutant f:Hex1 proteins that contained serines and threonines substituted in combination (f:Hex1(4A) and f:Hex1(4D)) or individually (f:Hex1(268D), f:Hex1(270D), f:Hex1(276D), and f:Hex1(278D)) were expressed in Jurkat cells and immunoprecipitated using anti-Flag agarose beads. The presence of P-TEFb in immunoprecipitations was followed by antibodies directed against CycT1. As expected, whereas wild-type and mutant f:Hex1(4A) proteins bound P-TEFb, the mutant f:Hex1(4D) protein failed to do so (Figure 5B, lanes 2–4). Interestingly, whereas the mutant f:Hex1(268D) and f:Hex1(276D) proteins did not alter these interactions, the mutant f:Hex1(270D) and f:Hex1(278D) proteins did not interact as potently (67% and 62% inhibition, respectively) as the wild-type protein with P-TEFb, suggesting that T270 and S278 are important for this interaction. Levels of Flag epitope-tagged proteins in our immunoprecipitations were similar (Figure 5B, lower panel). Moreover, individual alanine substitutions did not affect the binding of the mutant f:Hex1 proteins to P-TEFb (not shown). Additionally, in contrast to the wild-type and mutant f:Hex1(4A) proteins, the mutant f:Hex1(4D) protein failed to bind P-TEFb in the LC as determined by glycerol gradient sedimentation analyses (not shown; see below).

Next, these features were supported further by transcriptional assays in cells (Figure 5C). We used a classical DNA-tethering system, which consists of the plasmid reporter pG6TAR that contains six Gal4 DNA binding sites and the plasmid effector Gal4.CycT1. The recruitment of Gal4.CycT1 to the promoter results in the P-TEFb-dependent activation of transcription that is sensitive to the inhibition by HEXIM1 [28]. Indeed, the mutant f:Hex1(4A) protein inhibited the transcriptional activation by Gal4.CycT1 even more robustly when compared to the wild-type f:Hex1 protein (Figure 5C, lanes 1–3). In contrast, the mutant f:Hex1(4D) protein, which failed to bind P-TEFb, did not inhibit this transcriptional activation (Figure 5C, lane 4). Similar results were obtained with mutant f:Hex1(270D) and f:Hex1(278D) proteins. Also, levels of Flag epitope-tagged proteins were similar (Figure 5C, lower panel). Collectively, these results indicate that T270 and S278 in the TBD of HEXIM1 are important for its binding to P-TEFb in cells. Furthermore, they suggest that these residues could be phosphorylated by Akt, leading to the disruption of the LC and a subsequent release of the transcriptionally active P-TEFb.

### T270 and S278 in the TBD of HEXIM1 Are Targeted by the PI3K/Akt Pathway

**Figure 6:**
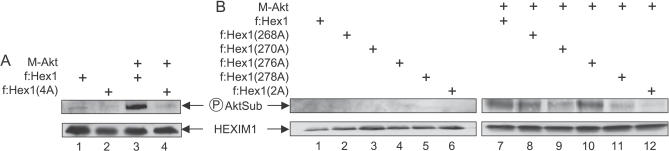
T270 and S278 in the TBD of HEXIM1 Are Phosphorylated following the Activation of Akt In Vivo f:Hex1 protein and mutant f:Hex1(4A) protein in (A), and mutant f:Hex1(268A), f:Hex1(270A), f:Hex1(276A), or f:Hex1(278A) proteins in (B) were expressed in Jurkat cells transfected with an empty plasmid vector or a vector expressing M-Akt. After 24 h, protein extracts were subjected to immunoprecipitation using anti-Flag antibodies, and levels of phospho-Akt substrates (pAktSub) and Flag-tagged expressed proteins were measured by western blotting.

To examine directly whether HEXIM1 is phosphorylated by Akt in cells, the wild-type and mutant f:Hex1(4A) proteins were expressed in the presence or absence of the mutant constitutively active form of Akt (M-Akt) in Jurkat cells. Cell extracts were subjected to immunoprecipitation as above using anti-Flag agarose beads. Western blotting with antibodies directed against phospho-Akt substrates revealed that the wild-type f:Hex1 protein was phosphorylated extensively only when co-expressed with the mutant M-Akt protein (Figure 6A, lane 3 and 6B, lane 7), whereas in its absence only a modest phosphorylation was detected (Figure 6A, lane 1 and 6B, top left panel). In sharp contrast, the mutant f:Hex1(4A) protein was not phosphorylated even in the presence of the mutant M-Akt protein (Figure 6A, lane 4). Importantly, whereas phosphorylation of the mutant f:Hex1(268A) and f:Hex1(276A) proteins was not affected, the phosphorylation of the mutant f:Hex1(270A) and f:Hex1(278A) proteins was significantly reduced (50% and 69%, respectively) when compared to the wild-type f:Hex1 protein (Figure 6, compare lanes 8 and 10 to lanes 9 and 11). Finally, when both T270 and S278 were substituted with alanines, which yielded the mutant f:Hex1(2A) protein, the phosphorylation was reduced even further (Figure 6B, lane 12). Also, immunoprecipitated Flag epitope-tagged proteins were detected at similar amounts (Figure 6A and 6B, lower panel). Taken together, these findings demonstrate that HEXIM1 is phosphorylated upon Akt activation in Jurkat cells and that the T270 and S278 are responsible for these effects. In addition, these results suggest that the phosphorylation of residues in the TBD of HEXIM1 via the PI3K/Akt pathway disrupts the LC.

### Conserved Threonines and Serines in the TBD of HEXIM1 Mediate Its Release from the LC

**Figure 7:**
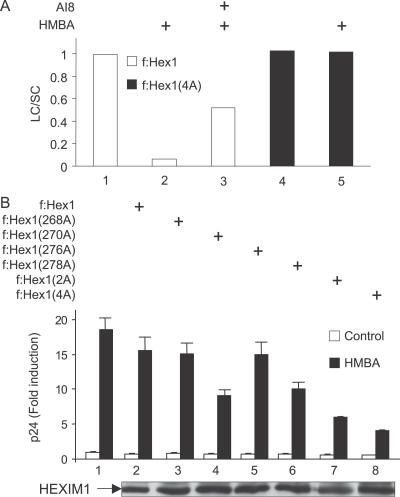
Mutation of the Conserved Threonines and Serines Renders HEXIM1 Insensitive to HMBA Treatment and Antagonizes the Induction of HIV Production by HMBA (A) Jurkat cells were transfected with plasmids expressing wild-type f:Hex1 or mutant f:Hex1(4A) proteins and then stimulated or not with HMBA (5 mM) for 1 h in the presence or absence of AI8 (1 μM). Total lysates were then subjected to glycerol gradient sedimentation and fractions were analyzed as in Figure 2. Western blot was performed using anti-Flag antibodies and the ratio LC/SC measured as in Figure 1. (B) U1 cells were transfected with an empty plasmid vector or those expressing mutant f:Hex1, f:Hex1(2A), f:Hex1(4A), f:Hex1(268A), f:Hex1(270A), f:Hex1(276A), or f:Hex1(278A) proteins. After 24 h, cells were stimulated or not with HMBA (5 mM), supernatant collected after another 24 h, and virus quantified using p24 ELISA.

To determine whether conserved threonines and serines were required for the HMBA-mediated disruption of the LC, we expressed the wild-type or the mutant f:Hex1(4A) proteins in Jurkat cells. As in Figure 1, the partitioning of f:Hex1 proteins in the LC and SC in untreated or HMBA-treated cells was followed by glycerol gradient sedimentation analyses (Figure 7A). Similar to endogenous HEXIM1 proteins, the wild-type f:Hex1 protein was released from the LC upon HMBA stimulation and the Akt inhibitor AI8 prevented this effect (Figure 7A, lanes 1–3). Critically, the mutant f:Hex1(4A) protein was not responsive to HMBA treatment and remained in the LC even after the stimulation (Figure 7A, lanes 4 and 5). Taken together, these results demonstrate that the conserved threonines and serines in the TBD play a major role in regulating the release of HEXIM1 from the LC upon HMBA stimulation of cells.

### HMBA Stimulation of Viral Production Depends on T270 and S278 in the TBD of HEXIM1

Finally, the use of the mutant f:Hex1(4A) protein that resisted the HMBA-mediated disruption of the LC enabled us to examine directly the importance of this phosphorylation for the stimulation of viral production. Wild-type or mutant f:Hex1(4A) proteins were expressed in U1 cells that were left untreated or treated with HMBA, and the production of new viral particles was measured as above. As presented in Figure 7B, the expression of the mutant f:Hex1(4A) protein antagonized effects of HMBA profoundly, as its induction of viral production was decreased by 75% (Figure 7B, lane 8). Consistently, individual substitutions of the threonine and serine at positions 270 and 278 with alanines reduced viral production by 50% and 40% in the presence of HMBA (Figure 7B, lanes 4 and 6), and mutation of both residues (f:Hex1(2A)) resulted in an inhibition comparable to that by the mutant f:Hex1(4A) protein (Figure 7B, compare lanes 7 and 8). In contrast, HMBA induced viral production robustly and the expression of the wild-type f:Hex1 protein had only a modest effect (Figure 7B, lanes 1 and 2). Also, levels of these Flag epitope-tagged proteins were similar as were basal levels of viral production in these cells (Figure 7B, lower panel and lanes 1–8). Taken together, these results indicate that HMBA induces viral production via the PI3/Akt pathway and the disruption of the LC via the phosphorylation of the T270 and S278 of HEXIM1.

## Discussion

In this study, we demonstrated that HMBA reactivates viral production in chronically infected cells via the transient release of active P-TEFb from the LC. These events depended on the transient activation of the PI3K/Akt pathway that led to the phosphorylation of T270 and S278 in HEXIM1. In turn, the released P-TEFb was recruited to the HIV promoter to stimulate transcription elongation. Importantly, the inhibition of the PI3K/Akt pathway and the transient expression of mutant HEXIM1 proteins that could not be phosphorylated and released from the LC via Akt antagonized the HMBA-mediated induction of viral production. Therefore, we provide a mechanism by which HMBA targets the pivotal cellular co-factor P-TEFb for the reactivation of HIV.

Thus far, a regulatory mechanism that releases P-TEFb from HEXIM1 and 7SK snRNA has not been determined. Here, we provide evidence that HMBA accomplishes this task by activating the PI3K/Akt pathway. Since inhibitors of both kinases and the expression of the mutant DN-Akt protein resulted in an identical phenotype, PI3K and Akt are involved in this release. Moreover, the mutant constitutively active M-Akt protein also released P-TEFb. It is attractive to hypothesize that HMBA, which is a bipolar compound, binds and activates PI3K directly or via aggregation. In such a scenario, the brief nature of this signal could reflect a rapid modification of HMBA and/or the sequestration and degradation of the signaling complex. The signaling pathway between PI3K and Akt and the subsequent nuclear translocation of Akt has been described in great detail elsewhere [36]. Since the mutant constitutively active M-Akt protein led to the phosphorylation of T270 and S278, it is tempting to speculate that Akt could phosphorylate these residues in the TBD of HEXIM1 directly. This phosphorylation also abrogated the binding between HEXIM1 and P-TEFb in cells. Indeed, this finding was supported by the analysis of mutant HEXIM1 proteins containing aspartic acids that mimic phosphorylated residues, which neither bound CycT1 nor inhibited P-TEFb. Notably, these residues are positioned in close proximity to the first coiled coil segment in the TBD, which is critical for CycT1 binding [26,37]. Thus, it is conceivable that the introduction of two negative charges by phosphorylation changed the structure of the adjacent first coiled coil segment, leading to the loss of binding between P-TEFb and HEXIM1 in cells. Finally, although the PI3K/Akt pathway also activates NF-κB [38], it is unlikely that it was responsible for the recruitment of P-TEFb to the HIV promoter in JΔK cells that lacked NF-κB-binding sites. Indeed, a previous study by Antoni et al. reached the same conclusion [15]. Rather, the recruitment of P-TEFb could have been achieved via other transcription factors that bind the HIV promoter, such as NF-AT, AP-1, LEF-1, and/or Brd4 [30,39].

Elucidating how HMBA mediates the disruption of the LC reveals one mechanism by which cells can regulate the release of active P-TEFb from HEXIM1 and 7SK snRNA. How other agonists such as stress-inducing agents and inducers of cardiac hypertrophy disrupt the LC remains unknown [23,30,33,34,40]. However, some of them could signal via receptor tyrosine kinases and other cellular kinases, i.e., they could also access the PI3K/Akt pathway. Moreover, our study also does not preclude that other RNA–protein and protein–protein interactions in the LC could be targeted via additional posttranslational modifications, which would also disrupt the LC [24,26–28,41].

A large body of evidence demonstrates that several different mechanisms can maintain HIV latency at the level of transcription (reviewed in [1,2]). These include changes in chromatin structure, transcription interference, insufficient levels of activators, P-TEFb, and/or the absence of the functional viral transcriptional transactivator Tat on the HIV promoter. Interfering with many of these mechanisms can reactivate latent HIV. Our study and those of others also suggest that this induction can be achieved by exposing latently infected cells to HMBA. Although HMBA is not an HDAC inhibitor, we found that it increases levels of P-TEFb on the HIV promoter, which led to the elongation of RNAPII that is accompanied by remodeling of the local chromatin structure. The disruption of the equilibrium between active and inactive P-TEFb by HMBA was transient, yet the resulting induction of HIV transcription persisted. This finding can be explained by the order of events that follow HMBA stimulation. During the first hours of HMBA treatment, the abundance of active P-TEFb leads to viral transcription in the absence of Tat. Since Tat releases P-TEFb from the LC by virtue of competing effectively with HEXIM1 and 7SK snRNA for binding to P-TEFb [42,43], its synthesis blunts effects of the reassembly of the LC. Thus, in the presence of Tat, HIV transcription continues despite modifications in the distribution of P-TEFb between the SC and LC.

How does our work contribute to a possible therapeutic use of HMBA and related bipolar compounds to purge the latent viral reservoir? Despite the fact that HMBA induced HIV transcription and production, we could not detect viral production in primary CD4+ T lymphocytes isolated from optimally treated individuals, most likely because this compound also interferes with the de novo infection ([16] and our results). Thus, HIV could not spread from a small number of infected cells (one in 10^6^ latently infected cells, [44]). However, we were able to demonstrate that HMBA could activate viral production in resting primary CD4+ T cells. Although these findings precluded the amplification of detectable viral particles, they represent yet another advantage for the potential therapeutic use of these agents. Thus, HMBA not only reactivates viral production but also inhibits new infection. Unfortunately, HMBA can not be used therapeutically as it is too toxic for human use. Alternatively, other bipolar compounds structurally related to HMBA might be tried, such as SAHA, which also activates the PI3K/Akt pathway [20]. Importantly, SAHA has been approved recently for the treatment of human cutaneous T cell lymphoma [45] and could thus be used alone or in combination with other therapies.

Finally, our findings might also shed a new light onto how modifications in the equilibrium between active and inactive P-TEFb complexes could affect cell growth, proliferation, and differentiation. HMBA induces the differentiation of many cells, including murine erythroleukemic and vascular smooth muscle cells, monocytes, lymphocytes, and neurons [46,47]. Importantly, HMBA also increases greatly the expression of HEXIM1 in vascular smooth muscle cells [22] and more recently, the transient disassembly and reassembly of the LC by HMBA was found to be associated with the differentiation of murine erythroleukemic cells [34]. Indeed, whereas the sustained activation of PI3K and Akt is associated with cell growth and proliferation [48], a transient activation of the PI3K/Akt pathway leads rather to cell differentiation and growth arrest. This seeming paradox can now be explained by the feedback mechanism involved in the regulation of P-TEFb. We propose that HMBA first disrupts the LC to release the active P-TEFb, which is followed by increased global transcription. In very short order, the LC reassembles with abundant new synthesis of HEXIM1 [22,34]. As the pool of active P-TEFb decreases, cellular reprogramming follows, leading to cell differentiation. As SAHA also activates transiently this pathway [20], the role of other bipolar compounds that are related to HMBA should also be investigated for their effects on PI3K and Akt.

## Materials and Methods

### Cell lines.

U1, ACH-2, and J1.1 cells were obtained through the NIH AIDS reagents program. JΔK cells were kindly provided by Arnold Rabson. Jurkat. U1, ACH-2, J1.1, and JΔK cells were grown in RPMI containing penicillin (100 IU/ml), streptomycin (100 μg/ml), and 10% FBS at 37 °C with 5% CO2.

### Plasmid DNAs.

Plasmid reporter pG6TAR and plasmids coding for the Gal4.CycT1 chimera or f:Hex1 protein were described previously [28]. To construct plasmids coding for the mutant f:Hex1 proteins, the pFlag-CMV-2. HEXIM1 plasmid was subjected to site directed mutagenesis with the QuickChange II XL Site-Directed Mutagenesis Kit (200521; Stratagene). The plasmids coding for myristoylated Akt (M-Akt) and dominant negative Akt (DN-Akt) were a gift from David Stokoe and were described previously [49].

### Immunoreagents and chemicals.

The anti-CycT1 (sc-8127) and anti-Cdk9 (D7) antibodies were obtained from Santa Cruz Biotechnology. The anti-HEXIM1 and anti-HEXIM2 antibodies were described previously [26]. The antibodies directed against the total (9272), phosphorylated form of Akt (9271), and phospho-Akt substrates (9611S) were obtained from Cell Signaling. DN-Akt and M-Akt were a gift from David Stokoe [49]. The anti-Flag M2 (F3165) antibody and the anti-Flag M2 beads (FlagIPT-1) were purchased from Sigma-Aldrich.

HMBA (Sigma-Aldrich) was resuspended in sterile water to obtain a stock solution of 1 M. Chemical inhibitors, Akt inhibitor VIII (AI8), and LY 294002 (Calbiochem), were resuspended in DMSO.

### Activation of HIV production in HIV chronically infected cell lines.

U1, ACH-2, or JΔK cells were plated at 2.10^5^ cells/ml in 24-well plates, supernatant was collected before stimulation with HMBA and then every day, and viral release in the supernatant quantified by p24 ELISA (PerkinElmer). Baseline levels were of 50 to 100 pg/ml in control cells. Pre-treatment with inhibitor was done 1 h before stimulation by HMBA. Plasmid transfection was done using Gene Pulser II Electroporator (Bio-Rad).

### Transient transfection and CAT reporter gene assay.

HeLa cells were seeded into six-well plates approximately 12 h prior to transfection and transfected with FuGENE6 reagent (1 815 091; Roche Applied Science). CAT enzymatic assays were performed as described [28]. Transcriptional activation of the pG6TAR reporter plasmid by the Gal4.CycT1 chimera was set to 100%. Error bars give standard errors of the mean.

### Immunoprecipitation assay and western blotting.

10 millions of Jurkat cells were lysed in 0.8 ml of lysis buffer A (10 mM Tris-HCl [pH 7.4], 150 mM NaCl, 2 mM EDTA, 1% NP-40, 0.1% protease inhibitor) for 0.5 h at 4 °C 20 h post-electroporation. The lysates were immunoprecipitated with the anti-Flag M2 beads for 2 h at 4 °C, washed extensively with the lysis buffer A, and the bound proteins were separated on SDS-PAGE electrophoresis. Western blotting was performed with the indicated antibodies and according to the standard protocols.

### Glycerol gradient sedimentation analysis.

Glycerol gradients (10%–30%) were established by pippeting 2 ml of each of the glycerol fractions (10, 15, 20, 25, and 30% v/v) in buffer A (20 mM HEPES [pH 7.9], 0.3 M KCl, 0.2 mM EDTA, 0.1% NP-40) into centrifugation tubes (Beckman), 331372. Gradients were formed by standing for 6 h at 4 °C. HeLa cells either transfected with the corresponding plasmids or not transfected were lysed in 0.5 ml of buffer A containing 0.1% protease inhibitor and either 0.5% RNase inhibitor or RNase A (100 mg/ml final concentration) for 30 min at 4 °C. The lysates were centrifuged at 10,000*g* for 10 min and the supernatants were loaded into tubes with the preformed glycerol gradients. Protein complexes were then fractionated by centrifugation in an SW 41Ti rotor (Beckman) at 38,000 rpm. for 21 h. Ten fractions (1 ml) were collected, precipitated with trichloracetic acid and finally analyzed by immunoblotting with the appropriate antibodies [25]. Films with exposures in the linear range were used and intensities of each bands were obtained using histograms with Photoshop. The ratio of proteins present in the LC (fractions 5, 6, and 7) versus the ones present in the SC (fractions 1, 2, and 3) was calculated. The ratio LC/SC was normalized to 1 for unstimulated cells.

### Infection of PBMCs and isolation of resting CD4+ T cells.

PBMCs were isolated from buffy coats of healthy HIV negative donors in a Ficoll density gradient (Pharmacia). PBMCs were then plated at 5.10^6^ cells per ml in 24-well plates, using RPMI 10% human serum AB. After 30 min, non-adherent cells (PBLs) were isolated and cultured in complete RPMI (containing penicillin [100 IU/ml], streptomycin [100 μg/ml], and 10% FCS) with IL-2 (10 U/ml) and PHA (3 ug/ml). After 3 d, cells were treated or not with HMBA (5 mM) in the presence or absence of Akt inhibitor 8 (AI8).

After isolation, PBLs (10^7^) were activated with PHA (5 μg/ml) and IL-2 (10 U/ml) for 3 d and infected with HIV-1_LAI_ (0.1 ng/ml of p24). They were cultured in RPMI, 10% FCS, supplemented with IL-2 (10 U/ml). After 11 d, resting CD4+ T cells were isolated by negative selection using magnetic beads (Invitrogen). Cells were more than 95% pure as assessed by FACS. These cells were then cultured in RPMI, 10% FCS. These cells expressed neither CD25 nor HLA-DR.

### ChIP assays.

ChIP was carried out essentially as described previously [50]. Cross-linking was achieved by incubating 70 million cells (J1.1 or JΔK) in 1% formaldehyde in medium for 10 min at room temperature. Cross-linking reactions were stopped by addition of glycine to a final concentration of 0.125 M. Cells were then pelleted in a conical tube and washed with cold phosphate-buffered saline. The cell pellets were then resuspended in 1 ml of Lysis buffer (1% SDS, 10 mM EDTA, 50 mM Tris-HCl [pH 8.0]) for 10 min on ice and subjected to sonication to obtain DNA fragments averaging approximately 200 to 500 bp in length. One-tenth of the total chromatin solution was used in each ChIP. Chromatin solutions were precleared with protein A/G-Sepharose beads and then incubated with the appropriate antibody at 4 °C overnight. Protein A/G-Sepharose beads were then added, and the mixture was incubated for another 2 h. The beads were washed five times in TSE-150 (0.1% SDS, 1% Triton X-100, 2 mM EDTA, 20 mM Tris-HCl [pH 8.0], 150 mM NaCl), TSE-500 (like TSE-150 but with 500 mM NaCl), and buffer III (0.25 M LiCl, 1% NP-40, 1% deoxycholate, 1 mM EDTA, 10 mM Tris-HCl [pH 8.0]), and twice in Tris-EDTA buffer. Immunocomplexes were eluted from the beads with elution buffer (1% SDS and 0.5% NaHCO3) for 15 min at RT. The DNA–protein complexes were then treated with proteinase K, followed by reverse cross-linking at 65 °C 4 h. DNA was extracted with phenol-chloroform, precipitated with ethanol, and dissolved in 30 μl of Tris-EDTA buffer. Two microliters of DNA was used with appropriate primer sets to amplify specific DNA fragments. qPCR was then performed using the Stratagene MX3000P real-time PCR system. Primers used for LTR were described previously [8] and primers for Gag span a region comprised between nucleotides 1144 and 1410. Standard curves for each primer pair was first obtained to check for their respective efficiency. Products were quantified using Brilliant SYBR Green QPCR (Stratagene) according to the manufacturer's directions. Relative intensity was calculated and normalized to the input. Fold-induction was assessed as the immunoprecipitation with specific antibodies over no antibody control.

## Supporting Information

### Accession Numbers

The National Center for Biotechnology Information (http://www.ncbi.nlm.nih.gov/) accession numbers for HEXIM1 are NM_006460 (nucleotide sequence) and BAA36166 (protein sequence).

## Abbreviations

Cdk9: cyclin-dependent kinase 9
ChIP: chromatin immunoprecipitation assay
CycT1: cyclin T1
HAART: highly active antiretroviral therapy
HDAC: histone deacetylase
HEXIM: HMBA-induced protein
HMBA: hexamethylene bisacetamide
LC: large complex
PBL: peripheral blood lymphocyte
PBMC: peripheral blood mononuclear cell
PI3K: phosphatidylinositol-3-kinase
PKC: protein kinase C
P-TEFb: positive transcription elongation factor b
RNAPII: RNA polymerase II
SAHA: suberoylanilide hydroxamic acid
SC: small complex
snRNA: small nuclear RNA
TBD: CycT-binding domain
